# Which ultrasound-guided parasternal intercostal nerve block for post-sternotomy pain? Results from a prospective observational study

**DOI:** 10.1186/s44158-023-00134-2

**Published:** 2023-11-16

**Authors:** Antonio Toscano, Paolo Capuano, Chiara Perrucci, Matteo Giunta, Alberto Orsello, Tommaso Pierani, Andrea Costamagna, Mario Tedesco, Antonio Arcadipane, Giuseppe Sepolvere, Gabriella Buono, Luca Brazzi

**Affiliations:** 1 Department of Anesthesia, Critical Care and Emergency, ‘Città della Salute e della Scienza’ Hospital, Turin, Italy; 2 Department of Anesthesia and Intensive Care, IRCCS-ISMETT, UPMC, 90127 Palermo, Italy; 3 Division of Cardiovascular Anesthesia and Intensive Care, Azienda Ospedaliera Ordine Mauriziano, Turin, Italy; 4 Department of Anesthesia and Intensive Care Unit and Pain Therapy, Mater Dei Hospital, Bari, Italy; 5 Department of Anesthesia and Cardiac Surgery Intensive Care Unit, Casa di Cura San Michele, Maddaloni, Caserta, Italy

**Keywords:** Fascial plane blocks, Parasternal blocks, Cardiac surgical procedures, Heart surgical procedure, Median sternotomy, Nerve blocks, Postsurgical pain

## Abstract

**Background:**

Parasternal intercostal blocks (PSB) have been proposed for postoperative analgesia in patients undergoing median sternotomy. PSB can be achieved using two different approaches, the superficial parasternal intercostal plane block (SPIP) and deep parasternal intercostal plane block (DPIP) respectively.

**Methods:**

We designed the present prospective, observational cohort study to compare the analgesic efficacy of the two approaches. Cardiac surgical patients who underwent full sternotomy from January to September 2022 were enrolled and divided into three groups, according to pain control strategy: morphine, SPIP, and DPIP group. Primary outcomes were was postoperative pain evaluated as absolute value of NRS at 12 h. Secondary outcomes were the NRS at 24 and 48 h, the need for salvage analgesia (both opioids and NSAIDs), incidence of postoperative nausea and vomiting, time to extubation, mechanical ventilation duration, and bowel disfunction.

**Results:**

Ninety-six were enrolled. There was no significant difference in terms of median Numeric Pain Rating Scale at 24 h and at 48 h between the study groups. Total postoperative morphine consumption was 1.00 (0.00–3.00), 2.00 (0.00–5.50), and 15.60 mg (9.60–30.00) in the SPIP, DPIP, and morphine group, respectively (SPIP and DPIP vs morphine: *p* < 0.001). Metoclopramide consumption was lower in SPIP and DPIP group compared with morphine group (*p* = 0.01). There was no difference in terms of duration of mechanical ventilation and of bowel activity between the study groups. Two pneumothorax occurred in the DPIP group.

**Conclusions:**

Both SPIP and DPIP seem able to guarantee an effective pain management in the postoperative phase of cardiac surgeries via full median sternotomy while ensuring a reduced consumption of opioids and antiemetic drugs.

## Introduction

Cardiac surgery techniques have seen rapid advancements over the past two decades; however, full median sternotomy remains the most common approach in cardiac surgery.

To avoid postoperative complications such as prolonged mechanical ventilation, mediastinitis, pulmonary infections [1], and chronic post-sternotomy pain [2, 3], adequate analgesic coverage in the postoperative phase is not only necessary but also recommended to ensure early extubation, mobilization, and discharge from the intensive care unit (ICU).

Unfortunately, the treatment of post-sternotomy pain is often inadequate, as it relies on opioids and other drugs (e.g., cyclo-oxygenase inhibitor, alpha-2 agonist, to provide additive and synergistic analgesic effect) providing minimal benefit to the patient and having significant adverse effects such as nausea, vomiting, ileus, respiratory depression, and sedation.

Therefore, a multimodal based on opioid-sparing analgesia strategies [4] and locoregional anesthesia techniques [5] have been proposed.

Neuraxial anesthesia—mainly thoracic epidural analgesia—and deep plexus blocks have shown to produce excellent analgesia and reduce systemic analgesic requirement [6], but in the cardiac surgery patients, anticoagulant and antiplatelet administration increase the risk of epidural hematoma classically related to neuraxial procedures [7]. Consequently, the American Society of Regional Anesthesia and Pain Medicine is still recommending a conservative approach to neuraxial techniques and other deep blockades, such as thoracic paravertebral block [8].

There is evidence suggesting that relatively new chest wall blocks named parasternal intercostal blocks (PSB) [9] could be effective alternatives for postoperative pain control in patients undergoing median sternotomy [10–13], even during antiplatelet and anticoagulant therapy [14].

Parasternal intercostal block can be achieved using two different approaches, the superficial parasternal intercostal plane block (SPIP) and deep parasternal intercostal plane block (DPIP), previously known as pectointercostal fascial block (PIFB) and the transversus thoracic plane block (TTPB), respectively [15]. Both techniques are effective in blocking the anterior cutaneous branches of the thoracic intercostal nerves (Th2–6) [16]. To date, in our facility, the use of locoregional anesthesia technique for median sternotomy is not considered a standard, due both to the lack of scientific evidence supporting its widespread use and for the learning curve of the US approach, which is fully mastered only by some clinicians.

Therefore, we designed the present prospective observational cohort study with the aim to compare the efficacy SPIP and DPIP with each other and with the standard treatment on postoperative pain relief in the first 48 postoperative hours after cardiac surgeries performed via medial sternotomy.

Our hypothesis was that PSB techniques would provide at least an equianalgesic effect compared to the standard opioid-based treatment, with less opioid consumption.

## Methods

The study has been approved by the local ethics committee (approval number 571/2021, December 25, 2021). All consecutive patients scheduled for cardiac surgeries performed via full median sternotomy between January and September 2022 at two different cardiac surgery centers (Città della Salute e della Scienza Hospital, Turin, Italy, and Azienda Ospedaliera Ordine Mauriziano, Turin, Italy) were included.

Exclusion criteria were age below 18 years, history of opioid abuse, and lack of informed consent. Written informed consent was obtained from all patients included in the study.

### Perioperative management

According to local perioperative protocols, general anesthesia was induced with intravenous (IV) midazolam, propofol, or etomidate, plus an IV opiate (Fentanyl or sufentanil, depending on the anesthetist’s choice). Neuromuscular block was achieved with induction bolus followed by continuous infusion of cisatracurium or rocuronium. Anesthesia was maintained by total intravenous infusion of propofol, and intraoperative analgesia was achieved by total intravenous infusion of sufentanil, tailored on patient’s heart rate, blood pressure, and bispectral index values.

Intraoperative care, including cardiopulmonary bypass (CPB), was managed in accordance with standard practice.

At the end of surgery, all patients were transferred to intensive cardiac surgical unit (ICU) for monitoring, respiratory weaning, and standard postoperative management. Sedation was maintained by propofol infusion for as long as deemed necessary. Postoperative pain was managed either with continuous intravenous (IV) morphine infusion or with a multimodal opioid-sparing strategy based on locoregional analgesia (SPIP or DPIP), according to the attending anesthesiologist choice, without interference.

Patients treated with IV morphine infusion received intravenous morphine (about 0.01 mg/kg/h as for standard practice in our center), starting from ICU arrival and titrated to clinical needs until ICU discharge.

In our facility, all the patients receive timed administration of acetaminophen (1 g every 8 h) for 48 h and rescue doses of morphine, tramadol, or ketorolac as needed to ensure pain control.

Both blocks (SPIP and DPIP) were performed at the end of surgery using ropivacaine 3 mg/kg diluted in a total of 60 ml of saline solution (10 ml per point, three points per side) for SPIP and in 40 ml total of saline solution (20 ml per side) for DPIP limiting the maximum dosage of ropivacaine to 300 mg.

### Ultrasound-guided superficial parasternal intercostal plane block (SPIP)

This block, originally described by de la Torre [16] in patients undergoing breast surgery, has been recently proposed by Kumar as an effective technique to reduce postoperative pain after sternotomy [10].

With the patient in supine position, after adequate skin disinfection, a linear ultrasound probe was placed on the chest in a parasagittal plane above the 2th, 4th, and 6th intercostal spaces, on the midclavicular line, 2 to 3 cm lateral to the upper third of the sternum. The intercostal spaces have been identified by counting the ribs through the probe.

**Fig. 1:**
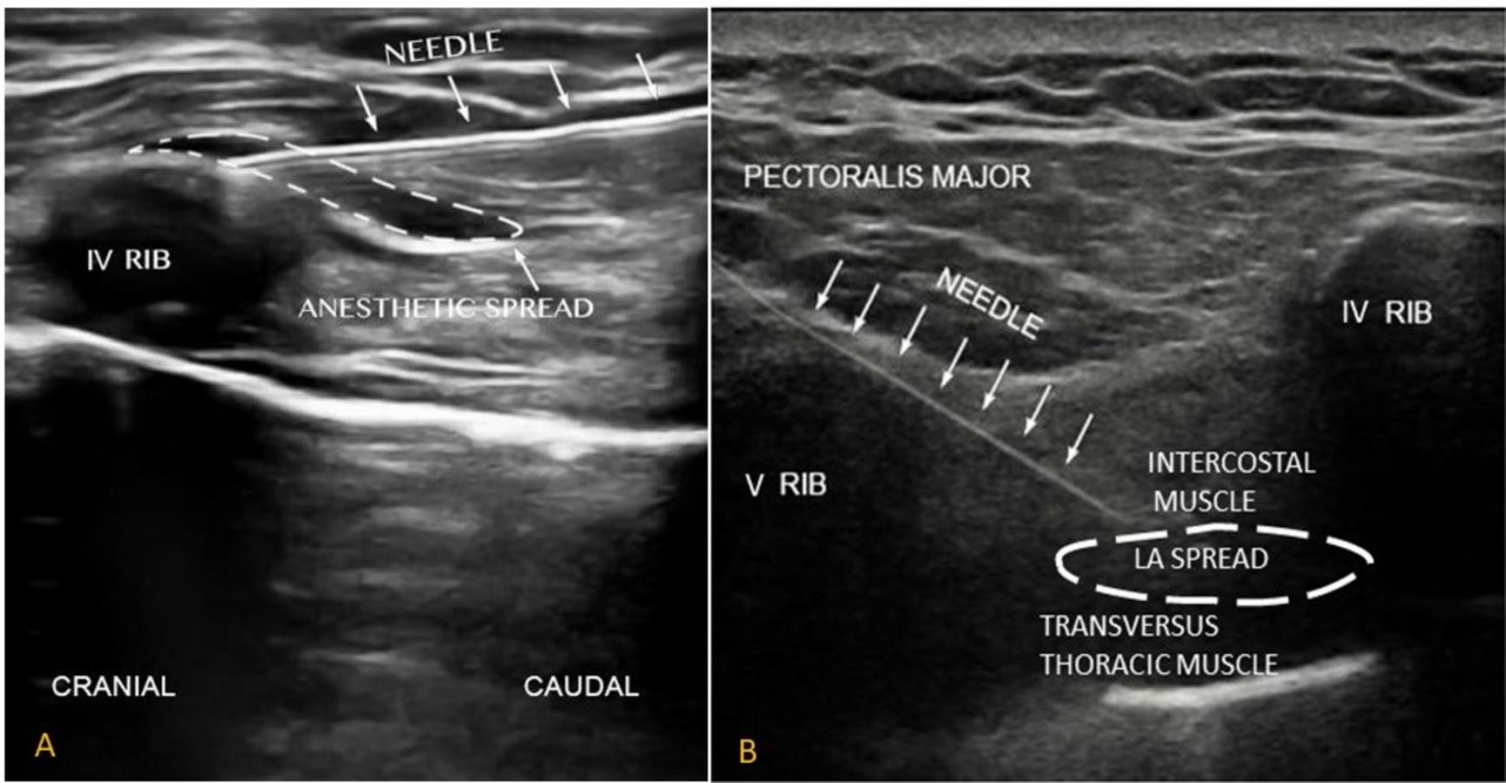
**A** Ultrasound-guided superficial parasternal intercostal plane block (SPIP). The tip of the needle reaches the interfascial plane between pectoralis major muscle and external intercostal muscle. The dotted area indicates the local anesthetic spread. **B** Deep parasternal intercostal plane block (DPIP). In the picture clearly visible, the tip of the needle between the interior intercostal muscle and transverse thoracic muscle and the anesthetic solution’s spread

A 22-gauge, 50-mm SonoPlex Stim needle (Pajunk Medical System, Tucker, GA, USA) was advanced via an in-plane approach from the cranial to caudal direction until it reached the interfascial plane between pectoralis major muscle and external intercostal muscle. After the position of the needle tip was confirmed, 10 ml of anesthetic solution was administered (Fig. 1A).

The same procedure was performed in the middle and lower one-third of the sternum and was repeated in the same way on the other side.

### Ultrasound-guided deep parasternal intercostal plane block (DPIP)

The DPIP is a deeper fascial plane block originally described by Ueshima [17] for breast cancer resection and used successfully for post-sternotomy pain in both adult [18] and pediatric patients [19].

Patient’s and probe’s position were the same as the SPIP. The maneuver was preceded by an assessment of the position of the internal mammary artery by color Doppler ultrasound to avoid accidental punctures of the vessel.

We used a 22-gauge, 50-mm SonoPlex Stim needle (Pajunk Medical System, Tucker, GA, USA) advanced in an in-plane approach from caudal to cranial direction until the tip of the needle was between the interior intercostal muscle and transverse thoracic muscles. After confirming the position of the needle tip and the correct plane with hydro dissection, 20 ml of anesthetic solution was administered (Fig. 1B).

### Data collection and analysis

Demographics characteristics, type and duration of surgery, timing and dosage of pain-related medications, and data regarding postoperative course were collected. Pain was assessed using number rating scale (NRS), and recovery from anesthesia was assessed using Richmond Agitation Sedation Scale (RASS) by attending physicians, who were not blinded to the perioperative analgesic choice. Both scores were collected at 3-h intervals in the first postoperative day and subsequently at least once a day. Recovery was classified “deep to moderate,” when RASS was below −3, or “light to no sedation,” when RASS was between −2 and +1. Nausea was evaluated using a scale from 0 to 3 (0 = absence; 1 = weak nausea; 2 = strong nausea; 3 = very strong nausea) according to local protocol, and bowel function was evaluated using a scale from 0 to 2 (0 = absence of bowel activity; 1 = gas; 2 = feces).

Postoperative morphine use was computed as cumulative dose including any infusion and/or rescue dose. If tramadol was used, we computed tramadol 100 mg equal to morphine 10 mg. The use of nonsteroidal anti-inflammatory drug (NSAID) as rescue therapy was computed separately.

### Study outcomes

The primary outcome was postoperative pain evaluated as absolute value of NRS at 12 h. Secondary outcomes were the NRS at 24 and 48, the need for salvage analgesia (both opioids and NSAIDs), incidence of mild adverse effects (i.e., nausea, vomiting, and incorrect catheter placement), quality (RASS), and timing of postoperative course (ICU and hospital length of stay, duration of mechanical ventilation starting from intubation in the operating room, ventilator-free days).

### Statistical analysis

According to a previous study [10] in which the median NRS score at 12 h in the control group was 3.5 (range 2.0 to 5.0) and expecting a 50% reduction of pain after SPIP and DPIP with a alpha error of 5% and power 90%, sample size was calculated to be 27 patients in each group.

However, considering potential dropout, a large number of patients was included during the study period.

Data were tested for normal distribution by Shapiro-Wilk test and are expressed as mean and standard deviation (SD) and mean with 95% confidence interval or median with interquartile range 25–75 (IQR), as appropriate. Data analysis was performed for parametric variables with test for independent sample. Kruskal-Wallis nonparametric ANOVA was used for nonparametric continuous variables, followed by Dunn-Bonferroni post hoc test with adjusted significance. Categorical variables were analyzed with Fisher’s exact test, as appropriate. Statistical analyses were performed using SPSS statistics software, version 27 (IBM). A *p*-value < 0.05 was considered statistically significant.

## Results

**Fig. 2:**
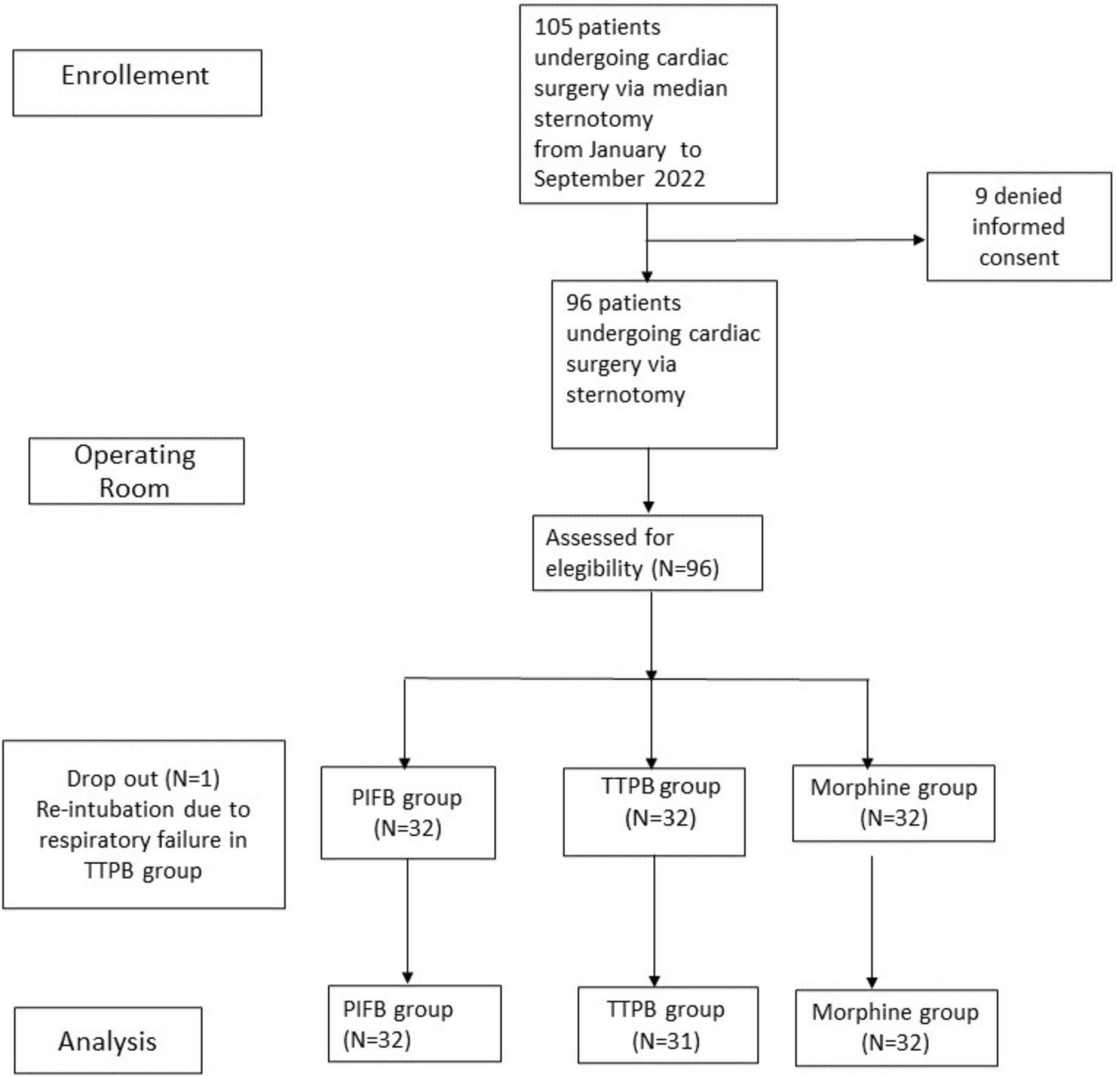
Flow chart

During the study period, 105 consecutive patients were evaluated for eligibility. Nine patients denied consent and were excluded, while the remaining ninety-six patients were enrolled. After data collection, 32 patients were assigned post hoc to the SPIP group, 32 were assigned to the DPIP group, and 32 to the morphine group. One patient in the DPIP group needed re-intubation due to type 1 respiratory failure 8 h after extubation and was therefore considered dropout and removed from the analysis, leaving 31 patients in the DPIP group (Fig. 2). According with the sample size described above, the enrollment started in January 2022 and was then interrupted in May 2022, having reached an adequate number of patients.

As shown in Table 1, there were no statistical differences between the three groups at baseline.

**Table 1:** Baseline characteristics of patients and surgery-related variables

Variables	Morphine group *n* = 32	PIFB group *n* = 32	TTPB group *n* = 31	*p*-value
**Age (years)**	62.23 ± 13.71	65.93 ± 12.74	64.38 ± 16.53	0.55
**Gender (M/F)**	20/12	23/9	20/11	0.70
**Weight (kg)**	72.65 ± 15.38	76.56 ± 10.40	72.29 ± 15.71	0.42
**Height (cm)**	167.84 ± 9.83	171.68 ± 9.46	169.87 ± 10.50	0.38
**BMI (kg/m**^**2**^**)**	24.95 (22.57–28.00)	26.50 (24.15–28.47)	25.80 (22.00–27.35)	0.47
**ASA**	3 (3–3)	3 (3–4)	3 (3–3)	0.33
**CPB (min)**	112.86 ± 39.09	123.38 ± 47.04	120.07 ± 49.95	0.67
**Clamp (min)**	87.00 ± 22.92	89.86 ± 24.54	82.4 ± 24.01	0.87
**CABG**	13 (40.6%)	10 (31.3%)	9 (28.1%)	0.47
**Sufentanil intra-op (μg)**	202.12 ± 69.58	202.29 ± 66.82	201.96 ± 73.08	0,65

*BMI*, body mass index; *ASA* score, American Society of Anesthesiologists physical status classification system; *CPB*, cardiopulmonary bypass; clamp, aortic clamp

Total postoperative morphine consumption was 1.0 mg (range 0.0 to 3.0 mg), 2.0 mg (range 0.0 to 5.50 mg), and 15.60 mg (range 9.60 to 30.0 mg) in the SPIP, DPIP group, and morphine group, respectively (SPIP vs morphine: *p* < 0.001; SPIP vs DPIP: *p* = 0.47; DPIP vs morphine: *p* < 0.001) (Table 2). Opioids used postoperatively as rescue doses were equal to 0.0 mg (range 0.0 to 0.0 mg) in the SPIP group and 0.0 mg (range 0.0 to 5.0 mg) in the DPIP group (*p* = 0.07).

**Table 2:** Outcomes measures

*Variables*	*Overall* *n = 95*	*Morphine group* *n = 32*	*PIFB group* *n = 32*	*TTPB group* *n = 31*
***NRS 9 h***	2.0 (0.0–3.0)	2.0 (0.0–2.25)	2.0 (0.0–3.0)	0.0 (0.0–4.0)
***NRS 12 h***	2.0 (0.0–3.0)	2.0 (0.0–3.0)	2.0 (0.0–3.0)	1.0 (0.0–3.0)
***NRS 24 h***	2.0 (0.0–2.0)	2.0 (1.0–2.0)	1.0 (0.0–3.0)	2.0 (0.0–2.0)
***NRS 48 h***	1.0 (0.0–2.0)	1.5 (0.0–2.0)	0.0 (0.0–2.0)	1.0 (0.0–2.0)
***Morphine total consumption (mg)***	5.0 (0.0–12.3)	15.6 (9.6–30.0)	0.0 (0.0–2.0)*	0.0 (0.0–5.5)*
***Morphine consumption 24 h (mg)***	4.8 (0.0–11.5)	14.8 (9.4–30.0)	0.0 (0.0–2.0)*	0.0 (0.0–5.0)*
***Morphine consumption 24–48 h (mg)***	0.0 (0.0–0.0)	0.0 (0.0–2.0)	0.0 (0.0–0.0)*	0.0 (0.0–0.0)
***Rescue dose***			0.0 (0.0–2.0)	0.0 (0.0–0.5)

*NRS*, numeric rating scale; morphine tot, total morphine consumption (continue infusion + rescue dose); morphine consumption 24 h, total morphine doses in the first 24 postoperative hours; morphine consumption 24–48, total morphine doses between 24 and 48 postoperative hours. Note: Results are presented as median (range 25–75). *p < 0.01 vs morphine group

Median NRS were 2.0 (range 0.0 to 3.0), 0.0 (range 0.0 to 4.0), and 2.0 (range 0.0 to 2.25) in the SPIP, DPIP, and morphine group respectively (*p* = 0.77) at 9 h; 1.0 (range 0.0 to 3.0), 2.0 (range 0.0 to 3.0), and 2.0 (range 1.0 to 2.0) in the SPIP, DPIP, and morphine group respectively (*p* = 0.98) at 12 h; 1.0 (range 0.0 to 3.0), 1.0 (range 0.0 to 2.0), and 2.0 (range 1.0 to 2.0) in the SPIP, DPIP, and morphine group respectively (*p* = 0.75) at 24 h; and 0.0 (range 0.0 to 2.0), 1.0 (range 0.0 to 2.0), and 1.0 (range 0.0 to 2.0) in the SPIP, DPIP, and morphine group respectively (*p* = 0.67) at 48 h.

NSAIDs use as rescue analgesia was not different among groups, while metoclopramide consumption was significantly lower in SPIP and DPIP group compared with morphine group (*p* = 0.01). There was no significant differences in the incidence of nausea and vomiting at 24 and 48 h and in the time to normal bowel function.

Mechanical ventilation lasted 420.0 min (range 300.0 to 525.0), 363.5 min (range 267.0 to 420.0), and 420.0 min (range 180.0 to 675.0) in the SPIP, DPIP group, and morphine group, respectively (*p* = 0.25).

Ventilator-free days (median 27—range 27 to 27) and length of ICU stay (median 1 day—range 1 to 1) were comparable between groups (Table 3).

**Table 3:** Secondary outcomes and other drugs consumption

*Variables*	*Overall* *n = 95*	*Morphine group n = 32*	*PIFB group* *n = 35*	*TTPB group* *n = 31*
**RASS 3 h**	−3.0 (−5.0–0.0)	−3.5 (−5.0 to 0.0)	−3.0 (−5.0 to 0.0)	−2.0 (−5.0 to 0.0)
**RASS 6 h**	0.0 (−1.0–0.0)	0.0 (−2.7 to 0.0)	0.0 (−0.25 to 0.0)	0.00(0.0–0.0)
**RASS 12 h**	0.0 (0.0–0.0)	0.0 (0.0–0.0)	0.0 (0.0–0.0)	0.0 (0.0–0.0)
**Nausea 24 h**	10 (10.5 %)	3 (30.0%)	2 (20.0%)	5 (50%)
**Nausea 48 h**	1 (1,%)	0 (62.5%)	0 (32.5%)	1 (100%)
**Bowel activity 24 h**	11 (11.5%)	4 (36.4%)	3 (27.3%)	4 (36.4%)
**Bowel activity 48 h**	30 (31.6%)	9 (30.0%)	9 (30.0%)	12 (40.0%)
**MV duration (min)**	360.0 (180.0–540.0)	420.0 (180.0–675.0)	420.0 (300.0–525.0)	363.5 (267.0–420.0)
**VFDs (days)**	27.0 (27.0–27.0)	27.0 (27.0–27.0)	27.0 (27.0–27.0)	27.0 (27.0–27.0)
**ICU discharge (days)**	1.0 (1.0–1.0)	1.0 (1.0–1.0)	1.0 (1.0–1.0)	1.0 (1.0–1.0)
**Ketorolac (mg)**	0.0 (0.0–0.0)	0.0 (0.0–0.0)	0.0 (0.0–00.0)	0.0(0.0–0.0)
**Metoclopramide (mg)**	0.0 (0.0–0.0)	0.0 (0.0–30.0)	0.0 (0.0–0.0)*	0.0 (0.0–0.0)*
**Ondasetron (mg)**	0.0 (0.0–0.0)	0.0 (0.0–0.0)	0.0 (0.0–4.0)	0.0 (0.0–0.0)

MV duration mechanical ventilation duration, time (min) between ICU admission and extubation; *VFDS*, ventilator-free days, 1 point [for] each day during the measurement period that [patients] are both alive and free of mechanical ventilation in the first 28 days; ICU discharge, time (days) between ICU admission and discharge; hospital discharge, time (days) between ICU discharge and hospital discharge. Note: Results are presented as median (range 25–75) and percentages, *p < 0.01 vs morphine group

No adverse effects directly attributable to SPIP technique were observed, while two pneumothorax related to the block occurred in the DPIP group. However, these two cases were treated with a conservative approach, leaving the patients spontaneous breathing and were then successfully discharged form ICU. No inhospital death was observed.

## Discussion

The main finding of this multicentric, prospective, observational study is that both the superficial parasternal intercostal plane block (SPIP) and deep parasternal intercostal plane block (DPIP) are able to guarantee adequate analgesia with obvious low opioids consumption and a reduction in antiemetics drugs consumption in the 48 h following open cardiac surgeries via full median sternotomy.

SPIP, given the same efficacy in controlling pain, appear safer than DPIP requiring a lower opioid rescue dose. Although both blocks anesthetize the same nerves (the anterior cutaneous branches of the thoracic intercostal nerves [16]), the injection site is completely different: SPIP requires two or three needle punctures on each side, while DPIP requires a single bilateral injection on the 4th/5th intercostal space.

In fact, in the context of parasternal region, the ultrasound imaging reveals a layered structure from the skin to the lung, which includes the following: soft subcutaneous tissues, major pectoralis muscle, exterior intercostal muscles, interior intercostal muscles, internal mammary artery, transversus thoracic muscle, and pleura.

Consequently, while DPIP allows the injection of local anesthetic closest to the anterior branches of the intercostal nerves, the SPIP need two or three injections to ensure an adequate LA spread in the fascial plane [9].

The SPIP, being more superficial, appears to be associated with fewer risks compared with DPIP, since transversus thoracic muscle is located closer to the pleura resulting in a greater risk of pneumothorax [20]. Another possible complication of DPIP is the lesion of the internal mammary artery that courses between the interior intercostal muscle and transverse thoracic muscle [21, 22]; this complication is however easily avoidable using the color Doppler ultrasound.

While there is evidence supporting the efficacy of SPIP and DPIP for the management of acute [11, 23–26] and chronic [27] post-sternotomy pain, very few studies compared these fascial blocks with other established method of sternotomy pain relief, and only one compared them among themselves [28].

In 2022, Kaya et al. [28] enrolled 39 patients in a double blind comparing the efficacy and safety of DPIP and SPIP. It was found that the two blocks had equal efficacy, in terms of opioid consumption, postoperative NRS, and length of ICU even if, surprisingly, they differed in the time to first rescue dose (280 min in the DPIP group vs 660 min in the SPIP group). Our findings are consistent with these results since the rescue opioid dose was found to be higher in the DPIP group.

It has also been hypothesized that the DPIP requires a greater learning curve compared with SPIP; the transverse thoracic plane is in fact deeper and closer to the pleura, and it could be more difficult to visualize the plane between the internal intercostal muscle and the transverse thoracic muscle. This could result in an increased risk of pneumothorax and unilateral spread of local anesthetic which, in turn, may explain the early and higher rescue opioids demand.

In our study, the postoperative course was similar in the different study groups. In particular, we observed no differences in the duration of mechanical ventilation, length of ICU stays, and/or postanesthesia recovery; this contrasts with the theoretical advantages underlying current opioid-sparing and enhanced recovery protocols [4].

Some elements can explain this lack of statistical significance and limited our findings. First, pain control in morphine group was better than expected and assumed to estimate the sample size. As a result, the study resulted retrospectively underpowered to confirm the observed difference. Secondly, the lack of randomization is a potential source of bias, and, thirdly, all study patients had a particularly short ICU stay justifying, in itself, a better postoperative course [29]. In addition, given the observational study design, PSB were performed by different anesthesiologist, with different skills and experience. Ultimately, failure to standardize intraoperative dosing of sufentanil may have played a role in postanesthesia recovery.

Postoperative nausea and vomiting (PONV) were similar in study groups. This is probably due to the multifactorial genesis of PONV, of which opioids are only one of the triggers. It is also known that in fast-track cardiac surgery, the incidence of PONV is relatively low. Prophylactic administration of antiemetic drugs is therefore usually not necessary [30].

## Conclusion

Although the use of traditional opioids is acceptable, both superficial parasternal intercostal plane block (SPIP) and deep parasternal intercostal plane block (DPIP) seem able to guarantee an effective analgesic coverage in the postoperative phase of cardiac surgeries via full median sternotomy while ensuring a reduced consumption of opioids and antiemetic drugs. Future studies are needed to confirm these preliminary results.

## Data Availability

The datasets used and analyzed during the current study are available from the corresponding author on reasonable request.

## Abbreviations

PSB: Parasternal intercostal blocks
SPIP: Superficial parasternal intercostal plane block
DPIP: Deep parasternal intercostal plane block
CPB: Cardiopulmonary bypass
ASA: American Society of Anesthesiology
ICU: Intensive care unit
NRS: Number rating scale
RASS: Richmond Agitation Sedation Scale
NSAID: Nonsteroidal anti-inflammatory drug
PONV: Postoperative nausea and vomiting
